# ABO blood group phenotypes influence parity specific immunity to *Plasmodium falciparum *malaria in Malawian women

**DOI:** 10.1186/1475-2875-6-102

**Published:** 2007-08-03

**Authors:** Edward Senga, Maria-Paz Loscertales, KEB Makwakwa, George N Liomba, Charles Dzamalala, Peter N Kazembe, Bernard J Brabin

**Affiliations:** 1 Department of Biochemistry, University of Malawi College of Medicine, Blantyre, Malawi; 2 Child and Reproductive Health Group, Liverpool School of Tropical Medicine, UK; 3 Baylor College of Medicine Children's Foundation, Lilongwe, Malawi; 4 Emma Kinderziekenhuis, Academic Medical Centre, University of Amsterdam, The Netherlands; 5 Department of Community Child Health, Royal Liverpool Children's Hospital, Alder Hey NHS Trust, Liverpool, UK

## Abstract

**Background:**

Blood group O has been significantly associated with increased placental malaria infection in primiparae and reduced risk of infection in multiparae in the Gambia, an area with markedly seasonal malaria transmission. This study analyses the association between ABO blood group phenotypes in relation to placental malaria pathology and birth outcomes in southern Malawi, an area with perennial malaria transmission.

**Methods:**

A cross-sectional study of 647 mother/child pairs delivering in Montfort Hospital, Chikwawa District between February-June 2004 and January-July 2005 was undertaken. Maternal peripheral and cord blood samples were obtained at delivery. Placental tissue was obtained and malaria histology classified as active, past or no malaria infection. Birth anthropometry was recorded. ABO blood group was measured by agglutination.

**Results:**

In primiparae, blood group O was significantly associated with increased risk of active placental infection (OR 2.18, 95% CI 1.15–4.6, p = 0.02) and an increased foetal-placental weight ratio compared to non-O phenotypes (5.68 versus 5.45, p = 0.03) In multiparae blood group O was significantly associated with less frequent active placental infection (OR 0.59, 95% CI 0.36–0.98, p = 0.04), and a higher newborn ponderal index compared to non-O phenotypes (2.65 versus 2.55, p = 0.007). In multivariate regression parity was independently associated with increased risk of placental malaria (active andpast infection) in primiparae with blood group O (p = 0.034) and reduced risk in multiparae with the same phenotype (p = 0.015).

**Conclusion:**

Parity related susceptibility to placental malaria is associated with the mothers ABO phenotype. This interaction influences foetal and placental growth and could be an important modifying factor for pregnancy outcomes. The biological explanation could relate to sialic acid dependent placental membrane differences which vary with ABO blood group.

## Background

In a previous analysis we reported for the first time an association between the ABO blood group phenotypes, placental malaria and pregnancy birth outcomes [1]. Blood group O was associated with an increased prevalence of active placental infection in primiparae and with a reduced risk in multiparae.

It is well established in non-pregnant subjects that the risk of acquiring *P. falciparum *infection with its associated complications is determined partly by host genetic factors, which include various haemoglobinopathies and red cell enzyme deficiencies [2]. Several studies have examined the effects of the ABO group phenotype on malaria risk in non-pregnant subjects and this evidence has recently been reviewed [3]. The limited data available which has examined clinical illness suggests that blood group A is a risk factor for severe malaria, whereas blood group O may offer some protection against severe disease. Epidemiological evidence in community studies supports this conclusion although comparison between studies is difficult because of design heterogeneity, as well as the variable malaria transmission in the different study locations.

Our previous analysis in Gambian pregnant women identified that the well established parity-related susceptibility to *P. falciparum *placental infection was associated with the blood group O phenotype [4]. That analysis utilised a historic data set which was made available from an early study in the Gambia, an area with seasonal malaria [5]. In the present study we sought to confirm if the association of ABO phenotype and malaria related outcome in pregnancy also occurred in women living under conditions of perennial malaria transmission in Malawi. We also wished to assess whether the magnitude of any association was comparable between pregnant populations in the Gambia and Malawi.

## Methods

### Study Population

The study was conducted between February-June 2004 and January-July 2005 at Montfort Hospital in Chikwawa District, Southern Malawi. Malaria is highly endemic in this area and transmission occurs year round, but it intensifies during the rainy season from December to April [6].

A total of 647 women were enrolled who attended the hospital for delivery during the study period. Women were approached during the first stage of labour and informed consent was requested for participation. A questionnaire was completed by a trained midwife requesting information on age, parity, obstetric history, bednet and antimalarial use. Women with emergency obstetric problems (toxaemia, haemorrhage, obstructed labour or sepsis) or with recent blood transfusion were excluded.

### Study Procedures

Maternal mid-upper-arm-circumference (MUAC) was measured at the mid-point of the left arm. A maternal peripheral and cord blood sample obtained after delivery was placed in an EDTA tube. Thick and thin blood smears were stained with Giemsa and screened for malaria parasites. ABO blood groups were typed by agglutination using commercial antisera (Biotech Laboratories Ltd., Ipswich, Suffolk, UK). Maternal and cord haemoglobin were measured by Haemacue (Haemacue Limited, Dronfield, Yorks, UK)

Babies were weighed following delivery using a balance scale to the nearest 10 gms, and crown-heel length was measured with a length board to the nearest mm. Gestational age was assessed using a modified Ballard score [7]. Placental weight was measured to the nearest 10 gms after large clots had been removed.

Following delivery of the placenta, a placental tissue sample was obtained by cutting a one cm cube of tissue from both the centre and peripheral placental cotyledons. These were fixed in 10% neutral buffered formalin. Biopsies were embedded in paraffin wax, sliced into thin sections and stained with Giemsa and/or haematoxylin and eosin. Slides were examined at the Histopathology Department, College of Medicine, Blantyre, by two observers, with discrepant findings assessed by a pathologist. Placental histology was classified according to the classification of Bulmer et al [8], which comprises non-infected, acute infection (presence of parasites without pigment); chronic infection (presence of parasites and pigment); past infection (parasites not present, pigment present).

Categorical variables were assessed with the chi-square or Fischer exact tests and odds ratios and their 95% confidence intervals estimated. Differences between means were assessed by ANOVA for normally distributed data or the Mann-Whitney/Wilcoxon test. Multiple linear regression was used to analyse factors associated with anthropometric outcomes. Factors with a significance level of p < 0.1 were included in the model. These were: maternal age, parity, mid-upper arm circumference and ABO phenotype. The babies' ponderal index was calculated from weight/length^3 ^(Rohrer's index).

## Results

The characteristics of the sample of women screened are summarised in table 1. Mean age was 25.8 years (range 15–49 years) and 31.8% were primiparae. Active *P. falciparum *placental malaria was present in 124 cases (19.5%), of which 96 were acute and 28 chronic. Past placental malaria occurred in 111 cases (17.4%). Compared to multiparae primiparae were at increased risk of active placental malaria (22.2% versus 18.0%, p = 5.00).

Table 2 shows the association of ABO phenotypes in relation to placental malaria category and parity. There was no association between the O blood group and risk of placental infection when parity was not taken into account. In primiparae, blood group O was significantly associated with increased risk of active placental malaria compared to non-O blood groups (OR 2.18, 95% CI 1.05–4.55, p = 0.02). In multiparae blood group O was significantly associated with less frequent active placental infection compared to non-O phenotypes (OR 0.59, 95% CI 0.36–0.98, p = 0.04). Blood group O was not associated with altered risk of past placental malaria in either primiparae (OR, 1.00, 95% CI 0.54–1.85) or multiparae (OR 0.82, 95% CI 0.46–1.43). In multivariate regression analysis parity was independently associated with increased risk of placental malaria (all categories) in primiparae with blood group O (p = 0.034), and reduced risk in multiparae for the same phenotype (p = 0.015).

**Table 1:** Maternal characteristics of study sample

Characteristics	n	Mean or %
Age (years ± SD)	647	25.8 (6.7)
Hb (g/dl ± SD)	619	11.5 (2.6)
MUAC (cms ± SD)*	646	25.9 (2.9)
Primiparae (%)	206	31.8
Blood group (%)		
A	136	21.7
B	161	25.7
AB	21	3.3
O	309	49.3
Placental infection (%)		
Active	124	19.5
Past	111	17.4
None	401	63.1

* Mid-upper arm circumference

**Table 2:** ABO phenotype by placental malaria category and parity

	Placental malaria		Phenotype N (%)				OR compared to non-O (95% CI)
		
	Type	No	A	B	AB	O	
Primiparae	Active	45	5 (9.8)	11 (22)	1	28 (29)	2.18 (1.05–4.55)
	Past	103	31 (61)	26 (52)	4	42 (44)	1.00 (0.54–1.85)
	None	55	15 (29)	13 (26)	1	26 (27)	0.58 (0.22–1.01)
	Total	203	51	50	6	96	
Multiparae	Active	78	21 (22)	22 (20)	5	30 (14)	0.59 (0.36–0.98)
	Past	56	12 (12)	14 (13)	5	25 (12)	0.82 (0.46–1.43)
	None	300	62 (65)	75 (68)	5	158 (74)	1.68 (1.12–2.52)
	Total	434	95	111	15	213	

When assessing the effect of parity on the risk of placental malaria, only O blood group mothers benefited from the protective effect of multiparity against placental malaria for active (primiparae vs multiparae, OR 2.51, 95% CI 1.34–4.70, p = 0.002), or active and past placental infection (primiparae vs multiparae, OR 3.83; 95% CI 2.31–6.36, p < 0.001). There was no protective effect of parity for active placental malaria for the other blood groups (non-O primiparae vs multiparae, OR 1.33, 95% CI 0.83–2.12, p = 0.2).

Birth outcomes by parity and maternal blood group phenotype, in relation to placental infection category, are summarised in Table 3. There were no significant differences by ABO category between O and non-O blood groups, for maternal or cord haemoglobin concentration, or birth length. Among multiparae, blood group O was associated with higher mean birthweight than for mothers with non-O groups (3098 g versus 3017 g, p = 0.05). When considering placental infection category, mean placental weight, was reduced in primiparae with the O phenotype with past or no placental infection, (p = 0.01).

**Table 3:** Mean birth outcomes by parity and maternal blood group phenotype

Parity	Placental Malaria	Blood group	n	Maternal Hb g/dl	Cord Hb g/dl	Birth weight g	Length m	Placental weight g	Ponderal index (‡)	Feto-placental weight ratio
Primiparae	Active	O group	27	11.16 (2.32)	14.50 (2.40)	2803 (460)	49.11 (3.27)	504 (94)	2.37 (0.37)	5.63(0.90)
		non-O	16	12.25 (3.56)	15.84 (3.39)	2747 (514)	48.18 (2.81)	510 (87)	2.43 (0.28)	5.38 (0.62)
	Past or no infection	O group	62	11.89 (3.03)	14.73 (2.64)	2811 (348)	49.03 (2.31)	498 (73)*	2.40 (0.32)	5.70 (0.76)†
		non-O	83	11.65 (2.18)	14.78 (2.27)	2843 (424)	48.62 (3.72)	526 (84)	2.54 (0.77)	5.46 (0.79)
Multiparae	Active	O group	28	11.39 (2.02)	13.99 (3.20)	3007 (476)	48.27 (3.24)	559 (107)	2.69 (0.47)	5.57 (1.29)
		non-O	46	11.04 (2.05)	14.11 (2.10)	2913 (540)	48.90 (3.43)	553 (113)	2.50 (0.53)	5.38 (1.16)
	Past or no infection	O group	178	11.49 (3.00)	14.45 (2.24)	3113 (434)	49.12 (2.83)	558 (81)	2.67 (0.70)	5.63 (0.85)
		non-O	170	11.29 (2.23)	14.08 (2.54)	3050 (395)	49.38 (3.04)	553 (81)	2.56 (0.46)	5.57 (0.65)

Brackets: standard deviation

Difference between blood group O and non-O *p = 0.01; †p = 0.055

‡ birthweight/length^3^

The ponderal index of babies born to multiparae with blood group O was higher than that for babies of non-O mothers (2.68 versus 2.55, p = 0.007). In multivariate regression including maternal MUAC, haemoglobin, parity, blood group and placental malaria in the model, only placental infection (F test 6.95, p = 0.009) and parity (F test 6.56, p = 0.01) were independent predictive factors for the babies ponderal index. Differences in ponderal index were associated to parity only in blood group O women.

Among primiparae the fetal-placental weight ratio was higher in women with blood group O (5.68 versus 5.45, p = 0.03). In multiparae the fetal-placental ratio was also increased in women with the O blood group, but this difference was not statistically significant (5.63 versus 5.53, p = 0.53). In multivariate analysis, parity (p = 0.03), and maternal haemoglobin at delivery (p = 0.03) were independent predictive factors for the fetal-placental weight ratio.

## Discussion

We have previously reported the association between increased prevalence of *P. falciparum *placental malaria infection in primiparae and reduced prevalence in multiparae in Gambian women with the blood group O phenotype. In the present study, in women from a high malaria transmission area of Southern Malawi, we observed a similar association, with a comparable risk estimate for the altered placental infection risk associated with active placental infection. In Malawian primiparae there was a 2.18 increased risk (95% CI, 1.05–4.55) of active placental malaria associated with blood group O, compared with a 2.99 estimate in the Gambia (95% CI 1.24–7.25). In multiparae the estimates for reduced risk of *P. falciparum *placental malaria (all histological types) in blood group O versus non-O were comparable between the two studies (0.45, 95% CI 0.21–0.97 in the Gambia, and 0.60, 95% CI 0.40–0.90 in Malawi). The similarity of these findings across two studies in different geographic locations in Africa, with a 40 year difference between the years when the studies were conducted, is striking. The histopathological assessments of placental infection category were also undertaken independently by different laboratory personnel. The studies in Malawi and the Gambia both showed increased mean fetal-placental weight ratios in multiparae with blood group O, compared to non-O phenotypes indicating that birthweight was increased relative to placental size. The consistency of these differences across the two studies with the associated improved placental malaria and infant anthropometric outcomes in multiparae with the O phenotype supports the conclusion of a parity-specific association of this blood group with protective malaria immunity.

In regions highly endemic for *P. falciparum *malaria red cell polymorphisms that confer resistance to malaria are widespread [9], and tolerance to malaria based on inherited factors is well described. Although several studies have analysed the association of ABO blood groups with malaria risk and severity in areas of different malaria endemicity, there have been no previous assessments of the role of the ABO blood groups in relation to placental malaria except for our previous analysis from the Gambia [1]. The nature of the ABO histo-blood antigens has recently been reviewed, and their population genetics and relationship to malaria infection in non-pregnant subjects is consistent with a pivotal role in governing infection risk, with a group O association with protective immunity [10].

Rosette formation in peripheral blood erythrocytes (i.e. binding of parasite infected erythrocytes to uninfected erythrocytes) has been associated with both the severity of clinical malaria in children and the blood group A antigen phenotype [11], but does not occur in placental malaria [12]. An in vitro study in fresh clinical isolates from the Gambia also confirmed that parasitized erythrocytes form rosettes more readily if belonging to group A or B than group O isolates [13]. Impaired rosette formation may thus contribute to the innate resistance to *P. falciparum *malaria that occurs in certain red cell disorders and in individuals of the blood group O phenotype. This could relate to enhanced parity specific immunity to placental malaria in women with the O phenotype.

Assuming incidence of *P. falciparum *infection is comparable across different parity groups, then enhanced parity-specific immunity in multiparae with the O phenotype must relate to improved clearance of placental parasites. Factors governing cytoadherence and placental parasite sequestration are likely to play an important role since infected erythrocytes express the unique variant surface antigen, VAR2CSA, which adheres to the glycosoaminoglycan chondroitin sulphate A expressed on the surface of syncitiotrophoblast [14]. *P. falciparum *alters constituents of the RBC membrane such as glycophorin band 1 and spectrin [15] contributing to antigenicity and clearance by the reticuloendothelial system. Some of these membrane proteins, including glycophorin A express ABO phenotypes. ABO phenotypes have also been found to be associated with the soluble endothelial cell markers and adhesion molecules [16].

In primiparae blood group O was associated with an increased fetal-placental weight ratio compared to mothers with non-O phenotypes. In multiparae with the O phenotype there was a higher newborn ponderal index, and fetal-placental weight ratio although the latter was not statistically significant. These findings are similar to the Gambian analysis which reported a significantly increased mean fetal-placental weight ratio in multiparae with group O, compared to non-O phenotypes (5.74 versus 5.36, p = 0.04). The consistency of these findings across the two studies suggests that the ABO interaction with placental malaria has an important effect on both placental and fetal growth. It remains to be clarified whether there is also a gestational age effect. In the Malawi study maternal HIV infection may have influenced fetal growth as maternal sero-positivity for HIV infection is reported at around 20% in this pregnant population [17]. For this reason and in view of the likelihood of other confounding factors, it is of interest that the effects on the fetal-placental weight ratios were comparable across the two studies. This supports the conclusion that the ABO histo-blood group antigens are of critical importance in the pathophysiology of placental malaria.

The practical implications of these findings relate not only to the recognition of particular mother-child risk groups, but also to the potential influence of these red cell antigens for both parasite replication and the immune response to malaria.
